# Talent Identification and Development in Male Futsal: A Systematic Review

**DOI:** 10.3390/ijerph191710648

**Published:** 2022-08-26

**Authors:** Diogo Mendes, Bruno Travassos, José M. Carmo, Felippe Cardoso, Israel Costa, Hugo Sarmento

**Affiliations:** 1 University of Coimbra, Research Unit for Sport and Physical Activity (CIDAF), Faculty of Sport Sciences and Physical Education, 3000-370 Coimbra, Portugal; 2 Research Center in Sports Sciences, Health Sciences and Human Development (CIDESD), Department of Sport Sciences, Universidade da Beira Interior, 6201-001 Covilhã, Portugal; 3 Portugal Football School, Portuguese Football Federation, 1495-433 Cruz Quebrada-Dafundo, Portugal; 4 Department of Physical Education, Centre of Research and Studies in Soccer (NUPEF), Universidade Federal de Viçosa, Viçosa 36570-900, Brazil; 5 Núcleo de Pesquisa e Estudos em Futebol, Universidade Federal de Viçosa, Viçosa 36570-900, Brazil

**Keywords:** expertise, indoor soccer, youth development, elite futsal

## Abstract

This review aimed to identify and synthesize the most significant literature addressing talent identification and development in futsal. A systematic review of Web of Science™ (all databases), Scopus, PubMed and SPORTDiscus databases was performed according to the preferred reporting items for systematic reviews and meta-analyses guidelines. This study included original articles in English, Spanish or Portuguese containing relevant data on talent development/identification of male futsal players. The search yielded 238 articles. After screening, a total of 32 manuscripts were fully reviewed. The quality of the data reviewed was generally good. Results indicate that the most successful players exhibit technical, tactical, anthropometric, physiological and psychological advantages and are distinguished from lower-level players based on their earlier dedication to training. This review highlights the need for those involved in the process of identifying and developing talented futsal players to consider the technical and tactical skills of futsal players, along with their anthropometric and physiological characteristics, scaled to age. Finally, special attention should be paid to the supportive psychosocial environments created in sports academies for developing futsal players. Overall, talent identification and development programmes in futsal need to be dynamic and offer the possibility of changing assessment parameters in the long term.

## 1. Introduction

Futsal has been gaining popularity worldwide, leading thousands of children to aspire to become futsal players, excited by the magnificent performances of top teams and motivated by exceptional players. Today, futsal has attracted many people’s interest due to the high skill level and agility of athletes [1]. Thus, scientific research on this sport has increased exponentially in the last decade to further understand the characteristics and specificities of futsal players and teams’ dynamics [2,3,4,5].

Generally, futsal requires players to perform high-intensity actions quickly and with little space, thus requiring high physical and physiological efforts, as well as tactical and technical skills. Moreover, during matches, the futsal players are expected to make quick decisions and swiftly perform offensive (passing, dribbling and shooting) and defensive (marking, intercepting balls) tasks [6]. Given the nature of futsal (such as the court size, game system, competition duration and skills involved), many variables influence success in this sport, and there is a need to identify the best selection criteria for identifying the best players [7].

In different sports, some players show exceptional qualities, which translate into extraordinary and consistent performances, allowing them to be designated as talents or claim that they have talent. For a long time, the talent identification process has comprised the detection, selection and development of players’ capacities conducted by experts [8]. Talent identification programs are designed to identify young athletes with the potential for achieving success at the senior elite level [9]. However, the use of the word “talent” does not always correspond to a clear understanding of how performances occur or the reasons that lead to these [10].

However, in futsal, like in similar sports (e.g., football), the idea persists that excellent players are born with a natural talent, which guarantees that they will have the attributes required to reach high levels of sporting expression [10]. For example, football players were traditionally selected by coaches based on a subjective analysis that recognized the potential of young players to complement the style of play of their club [11].

This conviction manifests itself in different forms and can be witnessed by the recurrent discourses and practices of various actors in sports. An innate understanding of talent can also be confirmed by an excessive concern with the so-called talent detection, through which the aim is to discover individuals who, at any given moment, exhibit above-average attributes.

One of the resulting problems is that most of the time, the appreciation of talent scouts is contaminated by side effects related to the biological age and physical precocity of players. That is, the older a child or young adult is relative to their teammates, the more likely they are to be considered gifted, even if they are not. This process has caused the negative discrimination of many players, as it does not identify the skills considered essential to become competent players during so-called capture sessions. The fact is ignored that the learning time and the consolidation of skills show high inter-individual variability, with different responses given by athletes to the stimuli to which they are submitted, specifically according to their biological and chronological age [12].

Scientific knowledge on talent identification and development in futsal has not progressed far enough to influence or inform sport practice. Despite increasing research interest, the best scientific approaches to successfully identify and develop futsal players remain unclear. Therefore, a systematic review of futsal and a synthesis of knowledge about the specificity of talent identification and development in this sport would help to better understand the game and evolve it even further.

Talented futsal players cannot be identified and developed reliably unless the skills that best match the specific demands of the game are identified. Thus, this article aimed to systematically review and organize the most significant literature addressing talent identification and development among male futsal players. The goal of this review is to identify the most frequently researched topics and characterize the methodologies used to study them.

## 2. Materials and Methods

### 2.1. Search Strategy: Databases and Inclusion Criteria

A systematic review of the available literature was performed according to the preferred reporting elements for systematic reviews and meta-analyses guidelines [13]. The electronic databases Web of Science (all databases), Scopus, PubMed and SPORTDiscus were searched for relevant publications using the keywords “futsal” AND “indoor soccer”. Each of these words was associated with the following terms: talent* OR expert* OR “talent identification” OR “talent development” OR “relative age effect” OR RAE OR “deliberate practice” OR “deliberate play”.

The publications included in the initial search met specific criteria. Specifically, they (1) contained relevant data concerning talent identification and/or development in futsal; (2) were performed on male futsal players; (3) were empirical studies; and (4) were written in English, Spanish or Portuguese. Studies were excluded if they (1) included practitioners from other sports, (2) did not contain any relevant data on talent development and/or identification, or (3) were reviews or conference proceedings.

Two reviewers (DM, HS) independently screened citations and abstracts to identify articles that might meet the inclusion criteria. Full-text versions of these articles were retrieved and independently screened by the same reviewers to determine whether they met the inclusion criteria. Disagreements regarding the eligibility of a study were resolved by consensus.

### 2.2. Quality of the Studies and Data Extraction

The overall methodological quality of the studies was assessed using the Critical Review Forms of Letts, Wilkins [14] for qualitative studies (with 21 items) and Law, Stewart [15] for quantitative studies (with 16 items). A data extraction form from the Cochrane Consumers and Communication Review Group data extraction template [16] was adapted to meet the requirements for inclusion of studies in this review and pilot tested on five randomly selected studies. Data were extracted independently by two authors (HS, DM).

## 3. Results

### 3.1. Search, Selection and Inclusion of Publications

**Figure 1:**
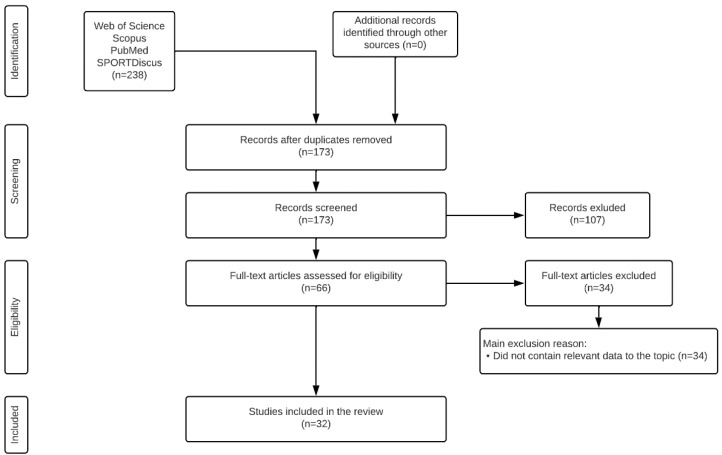
Flow chart of the article search procedures.

The initial search identified 238 titles, which were then exported to reference manager software (EndNote™ X9, Clarivate Analytics, Philadelphia, PA, USA). Sixty-five duplicates were eliminated either automatically or manually. The remaining 173 articles were then screened for relevance based on their titles and abstracts, resulting in another 107 studies being eliminated. The full texts of the remaining 66 articles were examined in more detail. During this process, 34 more articles were rejected for not meeting the inclusion criteria. Ultimately, 32 articles were selected for in-depth reading and analysis (Figure 1).

The main and unique factor for studies’ exclusion (*n* = 34) was their lack of relevance to the topic of the current review. The chronological analysis of the articles considered in this review evidenced recent developments in this area of research and highlighted that more than half of the studies (59.38%) were published in the last four years (i.e., since 2018).

### 3.2. Quality of the Studies

The most noteworthy results were that (1) the mean score for the 32 selected studies was 68.8%, (2) no publication scored below 50%, (3) only seven studies scored more than 80% and (4) 13 publications achieved an overall rating of more than 75%.

### 3.3. General Description of the Studies

The results were organized by categorizing the studies according to the major research topics that emerged from the content analysis based on a previous classification from the review published by Sarmento, Anguera [11] on male football players.

**Figure 2:**
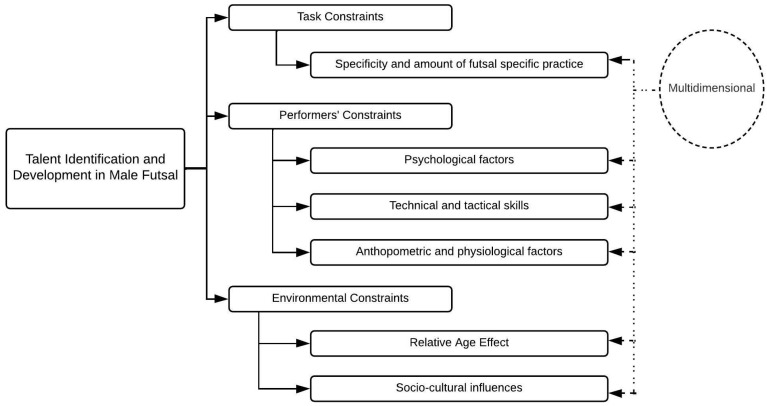
Scopes of talent identification and development in futsal.

Similar to the results of the systematic review of Sarmento, Anguera [11], the studies on male futsal players reviewed in the present work were markedly multidimensional [17,18,19,20]. Nevertheless, the generality was focused on a single topic. Namely, these topics were (1) task constraints: (a) specificity and volume of practice; (2) performers′ constraints: (a) psychological factors, (b) technical and tactical skills and (c) anthropometric and physiological factors (compared according to competitive level, playing positions and birth month); (3) environmental constraints: (a) relative age effect (RAE) and (b) socio-cultural influences; and (4) multidimensional analysis (Figure 2).

#### 3.3.1. Task Constraints

##### Specificity and Amount of Practice

Recent research indicates that elite players are distinguished from lower-level players for their earlier dedication to training and specifically to futsal (Table 1), as it is important to progressively increase the volume of weekly training [21].

#### 3.3.2. Performers′ Constraints

##### Psychological Factors

Six papers addressed information exclusively dedicated to the psychological factors affecting futsal players (Table 2). These investigations focused mainly on decision-making processes [18,20,47], electro-cortical activity and reaction time [25], the teaching–learning-training process [26] and self-confidence and stress control [23]. As such, these studies provide information about the psychological factors associated with career success in futsal. The results showed that goalkeepers had the best psychological profile. Pivots and wing-pivots had less self-confidence than other players, while universal players had less stress control than players in all other positions. Furthermore, players whose training centred on decision-making developed more than other players in term of intelligence and creative actions.

##### Technical and Tactical Skills

In general, coaches and scouts believe that athletes′ technical and tactical abilities are a strong predictor of future performance. This is shown in several studies (Table 3) which provide relevant information for talent identification systems. The results show that futsal training can develop players′ multifunctional athletes. The results also show that some individual skills like dribbling, passing or receiving the ball are characteristics of elite futsal players [27,28,29,30]. Such characteristics are certainly intrinsically related to the environment and task constraints of the sport. In addition, elite players spent more time during matches sprinting, whereas semi-elite players recorded higher percentages of standing and walking [27].

##### Anthropometric and Physiological Factors

As in other sports, anthropometric and physiological factors have been studied in relation to talent identification and development in futsal. The reviewed studies analyzed athletes’ body composition, aerobic power, abdominal strength endurance, flexibility, leg strength, lower limb power, speed and agility [32] using various tests such as the counter movement jump test, *t*-test and repeated sprint ability test (Table 4). The reviewed studies have shown that anthropometric characteristics and physical abilities do not necessarily make a difference between players in the post-pubertal period and should not be overestimated during early development [34]. In terms of somatotype, results showed that the endomorphic component was predominant in relation to the mesomorphic and ectomorphic components, irrespective of a team’s ranking at the end of a competition [35].

#### 3.3.3. Environmental Constraints

##### Relative Age Effect (RAE)

RAE has been widely researched as a factor that may influence selection and talent identification in team sports [50]. This review highlighted some literature already analyzed in futsal (Table 5). Studies have shown an RAE in male futsal, predominantly on athletes born in the first and second quartiles, highlighting that the RAE is an influencing factor in talent identification and development [39,40,41,42]. In the most recent study included in this review, conducted in Portugal, data showed an RAE in female and male youth soccer, as well as in futsal, particularly among young males and in clubs and academies with a high certification level, which could be accompanied by a loss of valuable elite players during the youth phase of their careers [36].

##### Socio-Cultural Influences

Despite the expected influence of the social environment on the identification and development of young athletes, only three studies exclusively have addressed this issue (Table 6). The results suggest that certain features of the built environment, such as indoor and outdoor play opportunities, may have less influence on talent development than previously thought [45]. The results also show that investment in an elite futsal career is critical to developing young athletes [48].

#### 3.3.4. Multidimensional Analysis

While most studies in this research area focused mainly on a single theme (Table 7 and Table 8), some multidimensional studies (Table 9) examined the effects of various factors on talent identification and development, including technical and tactical performance, anthropometric characteristics, the specificity and amount of futsal-specific practice and RAE (Figure 2). The results revealed that (1) athletes born in the first months of the year showed longer practice times than those born in the later months of the year, (2) players with low levels of knowledge successfully performed some easy technical and tactical skills like open passes and (3) anthropometric characteristics and isolated skills do not predict match-related technical performance in elite adolescent players and should be used with caution for talent identification [18].

A recent article described the predictors of selection in a youth academy, revealing that 30 m sprint speed was the best indicator, and that predictions were not improved when other predictors or their rates of change were included. Individuals selected by association at age 12 were predicted well above chance based on their sprint speed at age 9 or 10 [46].

## 4. Discussion

This article presented a review of the available literature on the talent identification and development of male futsal players. The results revealed four topics of analysis: task constraints, performers′ constraints, environmental constraints and multidimensional analysis.

In the following sections, we discuss some of the most interesting results that emerged from the analyses conducted in this review based on a theoretical framework of ecological dynamics.

### 4.1. Task Constraints

#### Specificity and Amount of Practice

There is abundant research on this topic in other sports. In the last 25–30 years, Ericsson, Krampe [52] and Ericsson [53] postulated that sport-specific, coach-led deliberate practice was the only type of activity that effectively leads to expert performance. They advocated maximizing deliberate practice, which required the early intensification and subsequent continuous expansion of deliberate practice. A widely held view is that 10,000 h of deliberate practice—highly structured activity with the specific goal of improving performance that require effort and are not inherently enjoyable [54]—are necessary and sufficient to reach the expert level. In contrast, the experiences of many elite athletes included various youth sports activities in both coach-led, organized training (e.g., in sports clubs, high-school sports or sports academies) and peer-led, non-organized activities in the athlete’s primary sport as well as in other sports. Some authors hypothesized that diversified experiences in youth may have favoured the later development of peak performance (e.g., Côté, Baker [55]; Davids, Güllich [56]).

One study included in our review indicates that elite players tend to start the practice of general sports and futsal earlier than lower-level players [21], revealing an interesting relationship between deliberate practice and the level of practice. Evidence from different sports demonstrates that early specialization is not the only way to reach expertise. Early diversification can also lead to elite performance, especially in sports in which performance peaks after biological maturity has been reached [57]. These two contrasting development pathway patterns—early specialization (deliberate practice) and early diversification (deliberate play)—have been discussed extensively in the literature [58]. Nevertheless, further studies are required in the context of futsal, as we found only one study on this topic in this sport.

### 4.2. Performers′ Constraints

#### 4.2.1. Psychological Factors

The study of psychological factors related to peak performance typically addresses two main questions: (1) “Which psychological skills are required to achieve top performance?” and (2) “How can these skills be developed in young athletes?” [58].

Our results suggest that the most successful athletes have high levels of decision-making [18,20,47], creative actions [26] and concentration and reaction time [25]. Goalkeepers showed the best psychological profile and characteristics, pivots and wingers had less self-confidence than average and universal players had less stress control than players in other playing positions [23]. These findings are useful for monitoring improvements in training and game performance and for identifying necessary changes in practice regimens.

The results reinforce previous results in other sports indicating that specific psychological abilities are necessary to become a high-level player [59], given that a player’s aptitude will exert either a positive or a negative influence on their technical–tactical and physical abilities [60]. Unfortunately, analyses of psychological aspects among futsal players are scarce, making it impossible to characterize the psychological abilities of futsal players [61].

In the field of soccer, the psychological profiles of Brazilian male and female professional soccer players were identified, along with their influence on the physical fitness of these players [62]. They concluded that certain psychological profiles (e.g., egocentricity, boldness, sensuality, insecurity, sensitivity, integrity and emotionality) could help players improve their performance in certain functions to solve problematic situations in the game, and that, there may be different responses to the same situation [63].

#### 4.2.2. Technical and Tactical Skills

Tactical skills are a player′s ability to perform timely actions to achieve a goal. Players with more expertise make better decisions [29]. Thus, the development of tactical skills seems crucial for achieving top-level performance in futsal.

Although only a few studies (*n* = 5) have addressed the importance of technical and tactical skills in identifying and developing talent in futsal, there is a clear link between high performance and superior technical skills, including ball receptions with the sole of the boot, ball driving, dribbling, passing and shooting [23,24,26,27]. Research has also highlighted that elite players are two-footed [27]. Studies have identified characteristics of elite futsal match-play that are closely related to the environmental and task constraints of the sport.

Similar to futsal, few studies in other sports have assessed sport-specific technical skills using a multidimensional approach. However, these skills predicted later performance in a range of sports, such as dribbling tests in field hockey [64]; swimming performance and ball-handling skills in water polo [65]; passing, dribbling, shooting and ball control skills in soccer [66]; and dribbling skills in handball [67]. In general, skill in futsal is based on several basic techniques—including passing, holding the ball, dribbling and shooting—which must be comprehended by every player [68]. Futsal players have to demonstrate these technical skills while being limited by space, the opponent and time [69].

Previous work on futsal has revealed that the spatial-temporal relations defined by the distances and angles between the ball carrier and the nearest defenders and by the distances and angles of defenders to the passing line are key pieces of information that guide players′ passing and shooting actions [70,71,72]. Nevertheless, no studies have clearly revealed how manipulating the environment of play constrains players′ learning. For this purpose, the ecological dynamics approach can offer theoretical guidance for coaches, sports scientists and practitioners to carefully design the microstructures of practice environments by manipulating task constraints [73].

#### 4.2.3. Anthropometric and Physiological Factors

Researchers have attempted to describe the physiological characteristics of futsal players at different levels of competition and at different ages [74]. For example, Alvarez, D’Ottavio [1] examined the aerobic fitness of futsal players of different competitive levels and found that VO2max could be a competitive-level-dependent physical variable in futsal. Pedro, Milanez [75], reported that running speed at the ventilatory threshold and maximal oxygen consumption discriminate the competitive level, with better performances exhibited by players with higher performance levels.

Furthermore, Nakamura, Pereira [76] investigated the differences in physical performance between Brazilian U-20 and senior top-level futsal players, and reported that long-term exposure to futsal can improve aerobic fitness and cardiac autonomic regulation while impairing muscular strength and speed performance. Recently, Sekulic, Foretic [77] highlighted the importance of agility in determining the performance level of professional futsal players by using newly developed tests of the change of direction speed and reactive agility in competitive futsal players.

Finally, the literature indicates that the performance levels of futsal players differ in terms of match running performance. Compared to sub-elite players, elite futsal players cover a greater total distance at higher intensities and perform more sprints during match-play [78].

Overall, studies analyzing the influence of anthropometric and physiological factors on talent identification reveal that elite players display better levels of body fat percentage, aerobic power, abdominal strength, speed performance and lower limb power and lower limb strength [32]. In terms of somatotype, the endomorphic component was predominant in relation to the mesomorphic and ectomorphic components, irrespective of a team’s ranking at the end of a competition [35]. In contrast, Ré et al. (2010) revealed that anthropometric characteristics and physical capacities do not necessarily differentiate players at post-pubertal stages and should not be overvalued during early development.

Empirical and theoretical literature indicates that it is important to identify specific performance characteristics for a development program that is supported by appropriate procedures for monitoring and recapture late maturers. This will help sports clubs understand the types of characteristics they can identify and develop in young players.

### 4.3. Environmental Constraints

#### 4.3.1. RAE

Several studies included in this review reported an RAE in female and male youth players from Portugal [36,39] and in male futsal players from Brazil [40,41,42,43]. However, a reverse RAE was observed in LNFS players from Spain [38]. These studies have shown that talent identification in futsal can be significantly affected by RAE, as coaches and scouts select the players who perform best at the time of selection, rather than the players who are the most long-term promise.

A better understanding of RAE in youth players would raise the awareness of the need to reduce bias due to (de)selection [36]. In general, a birthdate closer to the beginning of the year (e.g., in the first three months) is associated with an athletic advantage, resulting in an over-representation of athletes born early in the year [79,80].

A wider appreciation of the athletic triangle, which comprises coaches, parents and athletes, as well as factors beyond the physical should be considered regarding the RAE [81]. This is particularly the case as data have demonstrated that the RAE is evident within pre-pubertal age groups, among which maturity-related factors should not be a contributing factor [82]. Researchers have studied the RAE in various team sports such as soccer [83], basketball [84], rugby [85], handball [86,87] and cricket [88]. The RAE has been shown to be a potential source of talent selection bias in different modalities [87].

Pre-established models give special importance to physical and conditioning variables. This might partially explain why relatively old players seem to perform better in the selection process than young players, leading to a higher selection success rate. The RAE has been extensively explored among male soccer players, but little is known about it in other contexts, such as female players and futsal athletes. Despite the popularity of futsal and the media coverage of major competitions, little research has been done on it, especially when compared to soccer. Further studies should consider the simultaneous influence of more than one constraint (e.g., influence of maturational status, criteria used for the selection of the players, philosophies of the clubs, etc.) that may arise from different sources using both research methods (qualitative and quantitative) to improve the understanding of RAE in futsal.

#### 4.3.2. Socio-Cultural Influences

While it is well-accepted that several environmental factors influence the development of young athletes [89], few studies have addressed this topic exclusively in the context of futsal. Players who have experienced a fruitful social context and familial socialization facilitating ‘ball-kicking capitals’ tend to develop a habitus that is better suited for futsal than soccer [44]—birthplace effect. Similar to this, it is suggested that the characteristics of an athlete′s built environment, such as indoor and outdoor play opportunities, may be less influential in talent development than previously assumed [45]. A study on Brazilian female futsal players revealed four considerable points to consider in talent identification and development in futsal: diversified experiences in childhood, the initiation of kicking ball games practices, (c) systematic participation in futsal practices and (d) investment in an elite futsal career [48].

Additionally, a better understanding of family influence on talent development is needed—namely, what support parents can provide to their children as futsal players and how parents can support futsal players at key transition points in their careers [58]. At the macro-structure level, more attention could be given to the management of school activities and futsal clubs. Soccer associations should consider what activities in the school day are conducive to talent development.

Based on the available evidence, it appears that coaches and scouts should invest in players who are naturally strenuous, whose primary goal is personal performance improvement rather than pleasure and who often train without immediate reward—deliberate practice. They should search for players who have a strong ability to make good decisions, act creatively, are focused and can react quickly, which is just as important as superior technical skills. In terms of physiological constrains, there seem to be adaptative traits that come from better levels of body fat percentage, aerobic power, abdominal strength, speed, lower limb power and lower limb strength. Finally, they should not give importance to RAE in the context of futsal, to achieve a higher success rate in selection.

However, the research on this topic is not complete, as there are likely missing variables that future research could address, variables that may be more important than others, or athletes may compensate in one area when another skill is lacking between others.

This systematic review of this sport is an attempt to continue to better understand and develop the game.

### 4.4. Limitations

A limitation of this systematic review is that it does not include grey literature. Thus, relevant publications may have been overlooked.

## 5. Conclusions

Recently, research interest in the development and identification of youth players in futsal has increased. Based on the considerable number of studies on this topic reviewed in this paper, it was possible to identify the most frequently addressed topics in this area of research. These are (1) task constraints: (a) specificity and volume of practice; (2) performers′ constraints: (a) psychological factors, (b) technical and tactical skills and (c) anthropometric and physiological factors; (3) environmental constraints: (a) RAE and (b) socio-cultural influences; and (4) multidimensional analysis (Figure 2).

The main findings of this review were as follows. For task constraints, an important relationship was identified between deliberate practice and a player′s level of success. Regarding performers′ constraints, the most successful athletes express a strong capability to make decisions, take creative actions, concentrate and react quickly. They also exhibit superior technical skills and better levels of body fat percentage, aerobic power, abdominal strength, speed performance, lower limb power and lower limb strength. Nevertheless, anthropometric characteristics and physical capacities do not necessarily differentiate players at post-pubertal stages and should not be overestimated during early development, thereby reducing the bias of (de)selection. In terms of environmental constraints, talent identification in futsal can be significantly affected by the RAE. Thus, it is suggested that the characteristics of the built environment, such as indoor and outdoor play opportunities, are understudied—just one study presented.

Our results indicate a complex relationship between tactical, technical, anthropometric, maturational, physiological and psychological factors in futsal players. The complex interactions among these factors should help futsal coaches and fitness coaches objectively define how they identify talent and what training perspectives they use to promote a specific playing style.

Overall, talent identification and development programmes in futsal must be dynamic to provide opportunities for changing evaluation parameters in the long term. However, further studies are needed to improve knowledge on this research topic. For instance, longitudinal research needs to be done. In addition, future research should address the influence of genetic factors on the status of elite athletic. Developing a multidimensional analysis of how different elements interact to influence talent identification and development in futsal would also be worthwhile. Finally, further research is needed on the psychological and environmental aspects that influence talent development in futsal.

Additionally, future complementary studies should include an evaluation of the current position in male futsal regarding talent, like it has been done in other sports [90].

It is hoped that this study will contribute to continue the actual promotion of male futsal in the world.

## Figures and Tables

**Table 1:** Assessment of quantitative studies quality.

Study	Q1	Q2	Q3	Q4	Q5	Q6	Q7	Q8	Q9	Q10	Q11	Q12	Q13	Q14	Q15	Q16	
[21]	1	1	1	1	1	NA	1	0	1	1	1	1	0	1	1	1	86.7%
[22]	1	1	1	1	1	1	1	0	1	1	1	1	1	1	1	0	87.5%
[23]	1	1	1	1	1	1	1	0	1	1	1	1	0	1	1	0	81.3%
[24]	1	0	1	1	0	1	1	0	1	1	1	1	0	1	1	1	75.0%
[25]	1	0	1	1	0	1	1	0	1	1	1	1	1	1	1	0	75.%
[26]	1	0	1	0	0	0	1	0	1	1	1	1	0	1	1	0	56.3%
[27]	1	1	1	0	0	1	1	0	1	1	1	1	0	1	1	1	75.0%
[28]	1	0	1	1	1	1	1	0	1	0	1	1	0	1	1	0	68.8%
[29]	1	1	1	1	0	1	0	0	1	1	1	1	1	1	1	1	81.3%
[30]	1	1	1	1	0	1	1	1	1	1	1	1	0	1	1	0	81.3%
[31]	0	0	1	1	0	0	0	0	1	0	1	1	0	1	1	1	50.0%
[32]	1	0	1	1	0	1	1	0	1	0	1	1	0	1	0	0	56.3%
[33]	1	1	1	1	0	1	0	0	1	1	0	1	0	1	1	0	62.5%
[34]	1	0	1	1	0	1	0	0	1	1	1	1	0	1	1	1	68.8%
[35]	1	0	1	1	1	1	0	0	1	1	1	1	0	1	0	0	62.5%
[36]	1	1	1	1	1	NA	1	1	1	1	1	1	0	1	1	0	86.7%
[37]	1	1	1	0	0	NA	0	0	1	1	1	1	0	1	1	0	60.0%
[38]	1	1	1	1	1	NA	0	0	1	1	1	1	0	1	0	0	66.7%
[39]	1	0	1	1	1	NA	0	1	1	1	1	1	0	1	1	0	73.3%
[40]	1	1	1	1	0	NA	0	0	1	1	1	1	0	1	1	0	66.7%
[41]	1	1	1	1	0	NA	0	0	1	1	1	1	1	1	1	0	73.3%
[42]	1	1	1	1	0	NA	0	0	1	1	0	1	1	1	1	0	66.7%
[43]	1	1	1	1	0	NA	0	0	1	1	0	1	1	1	1	0	66.7%
[44]	1	0	1	1	1	1	1	1	1	0	1	1	0	1	1	1	81.3%
[45]	1	1	1	1	0	0	1	1	1	0	1	1	0	1	1	0	68.8%
[46]	1	1	1	1	1	1	0	0	1	1	1	1	0	1	1	0	75.0%
[17]	1	1	1	1	0	1	0	0	1	0	1	0	1	1	1	1	68.8%
[20]	1	1	1	1	1	1	0	1	1	1	1	0	0	1	1	0	75.0%
[18]	1	1	1	1	0	1	0	0	1	1	1	1	0	1	1	0	68.8%
[19]	1	0	1	1	0	0	1	0	1	1	1	1	0	1	1	0	62.5%

**Table 2:** Assessment of qualitative studies quality.

Study	Q1	Q2	Q3	Q4	Q5	Q6	Q7	Q8	Q9	Q10	Q11	Q12	Q13	Q14	Q15	Q16	Q17	Q18	Q19	Q20	Q21	
[47]	1	1	1	0	1	0	1	0	0	0	0	1	1	1	1	0	1	0	0	1	1	57.1%
[48]	1	1	1	1	1	1	1	0	1	1	0	0	1	1	1	1	1	0	0	1	1	76.2%

**Table 3:** Studies with predominantly specificity and volume of futsal-specific practice analysis.

Study	Sample	Main Variables	Procedure	Results	Quality Score
Pereira Ramalho Serrano, Leal dos Santos [21]	Study 1: 371 futsal players from three levels of expertise—elite (*n* = 60), medium (*n* = 160) and regional (*n* = 151) Study 2: 294 futsal players—24 from national team A and 270 amateurs.	Sport-initiation, early sport involvement and specialization in futsal training	Validated questionnaires from [49] which provides retrospective information about the sport activities experienced throughout athletes′ career.	Elite players are distinguished from lower-level competitive players for their earlier dedication to the sports training (specifically to futsal).	86.7%

**Table 4:** Studies with predominantly psychological factors analysis.

Study	Sample	Main Variables	Procedure	Results	Quality Score
Pastor-Vicedo, Prieto-Ayuso [22]	97 youth football players (from u-10 to u-14 age group), which 18 was identified—talent pool—and 1087 actions.	The process of decision-making	The Nomination Scale for Identifying Football Talent was utilized to screen the talent pool (*n* = 18), and the Game Performance Evaluation Tool was used for analyzing the 1087 actions completed.	The effectiveness must be more than 80% for children to be considered talented. A greater effectiveness of DMUs was shown in older age groups.	87.5%
Álvarez-Kurogi, Onetti [23]	167 young futsal players from 14 years old to 19 years old (candidates for the Spanish National Futsal Team, competing at the highest level in the U16 and U19 categories)	Psychological characteristics and their influence in performance	Questionnaires about their physical person (height, weight, age), their social-demographic characteristics as a futsal player and psychological profile.	Goalkeepers had the best psychological profile and characteristics related to SP. Pivots and wing-pivots had less self-confidence, and universals players, less stress control in relation to the rest of the playing positions.	81.3%
Bennett, Novak [24]	328 academy youth soccer players from three developmental stages: late childhood (8.0–10.9 year), early adolescence (11.0–13.9 year) and mid-adolescence (14.0–16.9 year).	Decision-making capacity	Video-based decision-making assessment, with response accuracy and response time recorded for various attacking situations.	Response times were significantly faster in early and mid-adolescent players when compared to those in the late childhood group. An overall decline in decision-making performance was observed from the 2 vs. 1 to the 4 vs. 3 situations.	75.0%
Duncan, Oppici [25]	23 male futsal athletes (11 players of an expert European futsal team and 12 amateur players-mean age: 28.7 ± 4.9 years).	Electro-cortical activity and reaction time	Congruent and incongruent trials of a modified Flanker task on a customized computer screen.	There was a significant difference in reaction time and error rate in congruent and incongruent task performance, and difference in electro-cortical activity between groups in the performance of both congruent and incongruent tasks.	75.0%
Moreira, Da Silva Matias [26]	30 futsal players aged 9 years from two different teams (15 players in each team).	Teaching-learning-training processes in tactical knowledge	Global analytical method.	Training methods lefted on decision making and development of tactical abilities are indicated to better promote the development of players capable of intelligent and creative actions.	56.3%
Ferreira Junior, de Almeida [47]	9 male sub-20 futsal players, aged between 17 and 20 years.	Decision-making capacity and declarative knowledge	Questionnaires about decision-making in variable situations.	The decision taken for the athlete and its justification, in some situations, were not ideal from the point of view of “experts”.	57.1%

**Table 5:** Studies with predominantly technical and tactical skill analysis.

Study	Sample	Main Variables	Procedure	Results	Quality Score
Yiannaki, Barron [27]	16 futsal players belonging to a national team during an international tournament, aged 25.74 ± 4.71 years.	Match performance: physical and technical performance.	Pre-tournament physical testing (Yo-Yo IR1) and match analysis (to select relevant technical variables for analysis. Data was generated using the SportsCode software package (v.11.2.15, Hudl, Lincoln, NE, USA).	Mean heart rate value during ‘court time’ of 164.7 ± 22.3 beats min-1, which as a percentage of participants′ MHR was 87.7% ± 4.4%, and a mean peak MHR of 98.3 ± 2.5%. Additionally, showed 2.16 ± 0.25 accelerating (>1.5 m·s^−2^) and 2.78 ± 0.13 decelerating (>1.5 m·s^−2^) events per player per minute. Match analyses showed that 77.3% of ball receptions were completed with the sole of the foot.	75.0%
Yiannaki, Carling [28]	77 participants were included, of which 56 were coaches from an “Advanced Youth Award” and 21 were players.	Futsal as a potential talent development modality for soccer	Surveys were used in order to gauge the perceptions of the participants.	89.6% of participants suggested that pitch size improved skills; 88.3% believed the pass back rule enhanced ball reception skills and; 89.2% perceived that futsal can help develop ‘multifunctional’ players.	68.8%
Práxedes, Moreno [29]	19 Spanish football players (U12), separated into two ability groups (Average versus Low skill-level).	Small-sided games on tactical behavior (decision-making capacity)	Game Performance Evaluation Tool (GPET).	Groups with an average level of expertise, training with numerical superiority in attack provides players with more time to make better decisions and to better execute actions.	81.3%
Mohammed, Shafizadeh [30]	144 elite players from 12 national teams and 60 semi-elite players from 5 domestic clubs in England.	Level of expertise	The matches were played again using Sports Code software (Sportstec, Co., Warriewood, NSW, Australia) that was configured in terms of hot key notation of actions in both physical and technical variables.	Elite players demonstrated more successful attempts in passing, dribbling and shooting. Elite players spent more time during a match on sprinting, whereas semi-elite players recorded higher percentages on standing and walking activities.	81.3%
Polidoro, Bianchi [31]	20 pre-teens 9–10 years-old organized by the coach in two homogeneous groups (sample group *n* = 10, control group *n* = 10).	Performance analysis and skills improvement–control of the ball, driving the ball and shooting	The players practice twice a week for one year. But only 10 players view training videos before each practice. The two groups have the same technical characteristics (homogeneous).	(1) The use of video-motor learning is more effective in the technical development of young players in the age of 9–10 years old; (2) A better acquisition of technical gestures by the kids’ part of the sample group in relation to the control group; (3) Video recording and reviewing gestures is a suitable tool for evaluating of motor execution; (4) Improved ability in self-assessment and self-correction.	50.0%

**Table 6:** Studies with predominantly anthropometric and physiological analysis.

Study	Sample	Main Variables	Procedure	Results	Quality Score
Da Silva Romero, Ferreira Da Rocha [32]	14 male futsal Brazilian athletes, aged 16 ± 1 years, participating in state championship under 20 category.	Morpho-functional profile	Tests to assess body composition, aerobic power, abdominal strength endurance, flexibility, leg strength, lower limbs power, speed and agility to change direction.	Futsal athletes showed body fat percentage classified as good (8.0 ± 3.3%) and higher aerobic power (VO2max: 59.7 ± 6.7 mL/kg/min). Abdominal strength endurance (45 ± 10 rep.) was rated as above mean, while flexibility was classified as mean (33 ± 6 cm). It was also shown suitable for speed performance (V30M: 4.8 ± 0.2 s), lower limbs power (221 ± 15 cm) and lower limb strength (101 ± 40 kgf).	56.3%
Galy, Zongo [33]	22 (14 Melanesian, aged 24.4 ± 4.4 years, and 8 Caucasian, aged 22.9 ± 4.9 years) elite futsal players.	Anthropo-metric and physiological characteristics	Tests of Counter Movement Jump, agility (T-Test), repeated sprint ability (RSA), RSA with change-of-direction (RSA-COD), sprints with 5 m, 10 m, 15 m and 30 m lap times, and aerobic fitness with the 30–15 intermittent fitness test (30–15 IFT).	Significantly lower height for MEL-G compared with NMEL-G and a trend for BMI. The jump-and-reach (CMJ) test showed significantly higher values for MEL-G compared with NMEL-G. The T-Test showed a significantly higher performance for MEL-G compared with NMEL-G.	62.5%
Ré, Corrêa [34]	49 Brazilian post-pubertal indoor soccer players (aged 16.9 ± 0.5 years old) of different competitive standards (24 elite and 25 non-elite).	Anthropometric characteristics and motor skills	Anthropometric measurements. Motor performance measurements: a toe kick with the preferred leg requiring maximal ball velocity and accuracy (defined target kick), a toe kick requiring only maximal ball velocity (undefined target kick), an agility running course with a ball, the same course without a ball and 15 m of linear running.	Anthropometric characteristics and physical capacities do not necessarily differentiate players at post-pubertal stages and should not be overvalued during early development.	68.8%
Queiroga, Ferreira [35]	112 female players (22.1 ± 5.4 years; 58.4 ± 6.9 kg; 161.8 ± 6.2 cm), from ten teams and seven different states, who had participated in Taça Brazil.	Somatotype and performance	The somatotype dispersion distance was applied to compare somatotypes between the champion team (reference) and the other teams (from 2nd to 10th position).	Endomorphic component was predominant in relation to the mesomorphic and ectomorphic component, irrespective of a team′s ranking at the end of the competition.	62.5%

**Table 7:** Studies with predominantly relative age effect analysis.

Study	Sample	Results	Quality Score
Figueiredo, Seabra [36]	Birthdates of 5306 female and 126,285 male soccer players, and 2437 female and 23,988 male futsal players (U7–U19), registered in Portugal during the season 2019/2020, and Portuguese National teams (from U15 to AA soccer teams and from U17 to AA futsal teams).	Data showed a RAE in female and male youth soccer and futsal, particularly in male, younger age categories, and in clubs and academies having a higher certification level, which could be accompanied by a loss of valuable elite players during the youth phase of their careers.	86.7%
Carraco, Galatti [37]	950 male futsal players who participated of the last three FIFA Futsal World Cups (2008, 2012 and 2016).	A mean CA of 27.76 ± 4.11 years with no differences for both playing positions and competitions. Elite male futsal athletes reach optimal competitive performance close to 27 years.	60.0%
Lago-Fuentes, Rey [38]	1873 professional futsal players who played in the First Division of the Spanish National Futsal League between seasons 2006–2007 and 2014–2015.	No homogeneous distribution was observed for any of the groups. Overrepresentation of players born during the last quarters of the year (Q3 and Q4), as well as a reduced presence of players born closest to the beginning of the year (Q1 and Q2). A RAE reversal was visible for LNFS players.	66.7%
Serrano, Shahidian [39]	2621 players, 220 adults international “AA” and 2401 young players of regional teams who participated in the inter-association national tournaments between 2014 and 2019.	Futsal in Portugal presents two completely different realities, with relative age effect being found in the male gender, but with no significant evidence in women. The inferential analysis of the male sample revealed this effect in: (a) the age groups under 15 (U-15), U-16 and U-17 years; (b) all competitive levels, except at the elite level; (c) all specific functions in the game, except “pivot”.	73.3%
Perondi, Dalla Valle [40]	848 futsal athletes, aged 23.18 ± 5.69 years—438 male athletes (aged 25.17 ± 6.11 years) and 410 female athletes (aged 21.06 ± 4.29 years)—who participated in Jogos Abertos de Santa Catarina, year 2015.	It was identified the effect of relative age in male futsal athletes while no relative age effect was identified in female futsal athletes. There is a prevalence of male athletes born in the first months indicating the existence of the RAE, whereas the RAE has not been verified in female athletes.	66.7%
Morales Júnior, Alves [41]	376 athletes from Brazilian Men National Futsal League (2013) and 227 from Brazilian Women National Futsal League (2014).	There is RAE in men futsal, predominantly on athletes born in the first semester. In female futsal, there is no evidence of RAE.	73.3%
Penna, Costa [42]	510 male futsal players from 23 Federação Mineira de Futsal teams (year 2009)-sub 11 (*n* = 90), sub 13 (*n* = 163), sub 15 (*n* = 103), sub 17 (*n* = 129), sub 20/senior (*n* = 125).	There were significant differences in the distribution of birth quartiles in four of the five categories evaluated, with a predominance of the first and second quartiles.	66.7%
Penna and Couto de Albuquerque Moraes [43]	370 professional players who played the Brazilian Futsal League in 2009.	Predominance of first and second quartiles, and an under representation of third and fourth quartiles. The RAE is a factor that could have an influence in player selection to futsal.	66.7%

**Table 8:** Studies with socio-cultural influences analysis.

Study	Sample	Main Variables	Procedure	Results	Quality Score
Marques, Schubring [44]	18 Brazilian National team futsal players.	Childhood socialization	Semi-structured interviews	The results show that (1) players experienced a fruitful social context and familial socialization that facilitated ‘ball-kicking capitals’, (2) developed a habitus that better matched the futsal than the soccer subfield and (3) delegitimized soccer as a suitable environment to participate in.	81.3%
van Nieuwstadt, Das [45]	1097 male football elite players from Netherlands and 243 male Dutch elite players.	Birthplace effect	Surveys	Characteristics of the built environment, such as indoor and outdoor play opportunities, may be less influential in talent development than previously assumed.	68.8%
Mascarin, Vicentini [48]	13 Brazilian women elite futsal players.	Career development	Retrospective, semi-structured, personal and individual interviews	Elite players demonstrated: (1) diversified experiences in childhood and youth, (2) initiation on ‘kicking ball games’ practices, (3) systematic participation in futsal practices and (4) the investment on elite futsal career.	76.2%

**Table 9:** Studies with predominantly multidimensional analysis.

Study	Sample	Main Variables	Procedure	Results	Quality Score
Fortin-Guichard, Huberts [46]	110 Dutch youth male football players aged 8–12.	Predictors of selection into a youth academy	Players were assessed on anthropometry, physical fitness, gross motor coordination, technical ability and psychosocial capacities.	The best indicator for predicting selection was the 30 m sprint speed. The individuals that the club ultimately selected at age 12 could have been predicted well above chance levels using the sprint speeds at age nine or ten.	75.0%
Ferreira, do Nascimento [17]	115 female futsal athletes, participants in the 20th Brazilian Cup of Adult Futsal Clubs.	Anthropo-metric profile and started status	Measurements of body mass, height, skinfold thickness, body perimeters and bone diameters, in order to characterize the anthropometric profile and determine the body somatotype.	There was no ERA in distribution, anthropometric profile, or starter status between athletes born from January to June and those born between July and December. On the other hand, those born in the first months of the year showed longer practice times than those born in the latter months of the year.	68.8%
Zuaneti Martins, dos Reis [20]	69 female futsal players, with a mean age of 23.75 ± 3.93 years old, members of their national teams (Bolivia, Peru, Uruguay, Colombia, Argentina and Venezuela).	Sports career and the formation context–practice start, amount of training and psychosocial support	Survey divided in 4 points: (a) place and age of beginning of futsal practice; (b) age at the beginning of systematic practice and investment in futsal specialization; (c) psychosocial elements to support futsal practice; (d) elements of dedication to training and career in the current stage of futsal.	Results showed a paradoxal context, in which the primary introduction to futsal occurred at the sports club, a structured place, but without sports competitions, to promote a development of the talent. It was also found that the majority began and first competed in futsal during adolescence, and their family members were their main motivators.	75.0%
Da Silva, Travassos [18]	80 passes performed by 40 male players differentiated by levels of experience, knowledge and skill.	Effect of experience, knowledge and skill on the performance	Questionnaire about the participants′ time and type of experience, Instrument of Evaluation of Declarative Tactical Knowledge and Game Performance Assessment Instrument [51].	Players with low levels of knowledge performed successful passes with higher initial angular values than unsuccessful passes. Experienced and skillful players completed successful passes based on information of the variability of emergence of angular interpersonal coordination.	68.8%
Re, Cattuzzo [19]	60 elite adolescent players (aged 14.0 ± 0.93 years) grouped as starters (*n* = 21), non-starters (*n* = 21) and substitutes without participation in the games (*n* = 18).	Anthropo-metric characteristics vs technical performance	Weight, height and skinfold thickness of the thigh, triceps and abdomen were measured following the standardization suggested by Lohman, Roche and Martorell (1988).	In elite adolescent players, anthropometric characteristics and isolated skills do not predict match- related technical performance and should be used with caution for talent identification or training purposes.	62.5%
